# Screening Autoxidation Propensities of Drugs in the Solid-State Using PVP and in the Solution State Using N-Methyl Pyrrolidone

**DOI:** 10.3390/pharmaceutics15030848

**Published:** 2023-03-05

**Authors:** Jayant Iyer, Anjali Karn, Michael Brunsteiner, Andrew Ray, Adrian Davis, Isha Saraf, Amrit Paudel

**Affiliations:** 1 Research Center Pharmaceutical Engineering GmbH (RCPE), 8010 Graz, Austria; 2 New Modalities and Parenteral Development, Pharmaceutical Technology & Development, Operations, AstraZeneca, Macclesfield SK10 2NA, UK; 3 Pfizer Worldwide Research and Development, Sandwich, Kent CT13 9NJ, UK; 4 Institute of Process and Particle Engineering, Graz University of Technology, 8010 Graz, Austria

**Keywords:** BDE, N-methyl pyrrolidone, RapidOxy^®^, autoxidation screening

## Abstract

Oxidative degradation of drugs is one of the major routes of drug substance and drug product instability. Among the diverse routes of oxidation, autoxidation is considered to be challenging to predict and control, potentially due to the multi-step mechanism involving free radicals. C–H bond dissociation energy (C–H BDE) is evidenced to be a calculated descriptor shown to predict drug autoxidation. While computational predictions for the autoxidation propensity of drugs are both swift and possible, no literature to date has highlighted the relationship between the computed C–H BDE and the experimentally-derived autoxidation propensities of solid drugs. The objective of this study is to investigate this missing relationship. The present work is an extension to the previously reported novel autoxidation approach that involves subjecting a physical mixture of pre-milled polyvinyl pyrrolidone (PVP) K-60 and a crystalline drug under high temperature and pressurized oxygen setup. The drug degradation was measured using chromatographic methods. An improved trend between the extent of solid autoxidation and C–H BDE could be observed after normalizing the effective surface area of drugs in the crystalline state, pointing to a positive relationship. Additional studies were conducted by dissolving the drug in N-methyl pyrrolidone (NMP) and exposing the solution under a pressurized oxygen setup at diverse elevated temperatures. Chromatographic results of these samples indicated a similarity in the formed degradation products to the solid-state experiments pointing to the utility of NMP, a PVP monomer surrogate, as a stressing agent for faster and relevant autoxidation screening of drugs in formulations.

## 1. Introduction

Stability testing ensures the safety, efficacy, and quality of the developed pharmaceutical product. Considerable time and effort are invested in the process of generating the stability data [1]. This data forms a part of the chemistry, manufacturing, and controls (CMC) dossier required for regulatory submissions, which is of importance for the licensing and approval of the finished product [2]. Potential instabilities (such as drug degradation) either in the drug substance or in the finished drug product may lead to shortcomings such as delays in the regulatory approval process, and if identified at late stages, may have undesirable consequences (e.g., market withdrawal/recall) with a potential waste of the time and efforts [3]. Against this background, the pharmaceutical industry has been continually striving to reduce the likelihood of such stability issues by performing accelerated stress testing/forced degradation experiments early in the drug development stage [3,4].

Forced degradation (or stress) studies provide an idea of the intrinsic stability of the drug and are intended to predict the likely degradation products (DPs)/mechanisms [5]. In addition, the stress samples can also serve as a reference for stability-indicating method development [6,7]. The accelerated stability test on the finished drug product must take batch variations, processing, packaging, and formulation factors into account. Among the different types of drug degradations, autoxidation has been a challenging mechanism to predict, elucidate, and control for several reasons [8,9]. First, oxygen is ubiquitous in the environment, and its levels cannot be readily limited in a manufacturing unit/environment. Second, the process by which an interaction of oxygen with solid drugs causes oxidation currently needs to be better understood [8]. Moreover, autoxidative stress methods in solids are relatively scarce, and the selection of antioxidants is still generally empirical [10]. Third, many factors leading to drug oxidation (presence of free radicals, moisture, heavy metals, etc.) can confound the degradation kinetics [11]. Finally, oxidative degradation products can be carcinogenic and toxic, and a small level of impurity can markedly limit the shelf life of a product [12,13]. In the context of the computational methods applied to predict the autoxidation propensity of drugs, the C–H bond dissociation energy (C–H BDE) is considered to be a useful descriptor [14,15,16,17]. This is because the autoxidation of drug molecules is shown to initiate by the abstraction of an H–atom, and is therefore often reaction-rate limiting [8,18].

While many oxidation methods are available in the solution state (using oxygen headspace [19], free-radical initiators [20], metals [21], peroxides [22], electro-oxidation [23], etc.), their applicability in the solid state has been limited [24,25,26]. The reason is that a drug molecule in the solution state can behave differently from that observed in the solid state due to enhanced molecular mobility, solvation, and kinetics. Furthermore, solid-state factors such as particle size, surface area, conformation/steric factors, hygroscopicity, surface chemistry, etc., may result in varied drug degradation [27]. An essential criterion for developing a rational forced degradation approach in the solid state is linked to how closely it can represent the degradation observed in a typical formulated product. Our recent article has evaluated the potential of using a commonly used azo-free-radical initiator (in the solution state), 2,2′-azobisisobutyronitrile (AIBN), to generate autoxidation in the solid state [28]. It was reported that AIBN decomposed at elevated temperatures from the solid matrix resulting in negligible drug degradation. On the contrary, using a pre-milled polyvinylpyrrolidone (PVP) excipient combined with the drug exposed under elevated temperature and oxygen pressure was shown to lead to marked autoxidation of the drug in the solid state. A point of importance here was that the DPs formed (in the solid samples) were identical to those generated in AIBN stress (solution) [28].

The present study investigates the subsequent applicability of the previously reported solid autoxidation approach to generate autoxidation products in a diverse set of solid drugs. While C–H BDE has been suggested to be a good descriptor to calculate autoxidation propensities, to the best of the authors’ knowledge, no works have investigated the relationship between this computed value and the experimental autoxidation rates for a set of compounds. It is in this context that the relationship between the C–H BDE and the autoxidation of the drugs in the solid state is probed. In parallel, the autoxidative reactivity of selected drugs was evaluated in the solution state by employing the monomer of the PVP excipient i.e., N-methyl pyrrolidone (NMP). NMP produces a hydroperoxide when heated in the presence of oxygen via a free radical autoxidation mechanism [19]. NMP is a valuable alternative for studying the miscibility of crystalline drugs in povidone excipients due to the structural similarity and molecular interaction (H-bond formation) [19,29]. Such miscibility studies help develop physically stable amorphous solid dispersions [30]. Also, due to substantially large and nearly planar non-polar groups in NMP, hydrophobic interactions with the drug can generate a stable complex. This association stabilizes the drug in dissolved form, making NMP a suitable oxidizer [31]. It is proposed that NMP as a surrogate to PVP could be helpful to enrich the autoxidation of drugs, and this autoxidation method can be expected to represent a rational strategy for evaluating autoxidation propensities of solid formulations comprising PVP as an excipient.

## 2. Materials and Methods

### 2.1. Materials

**Figure 1:**
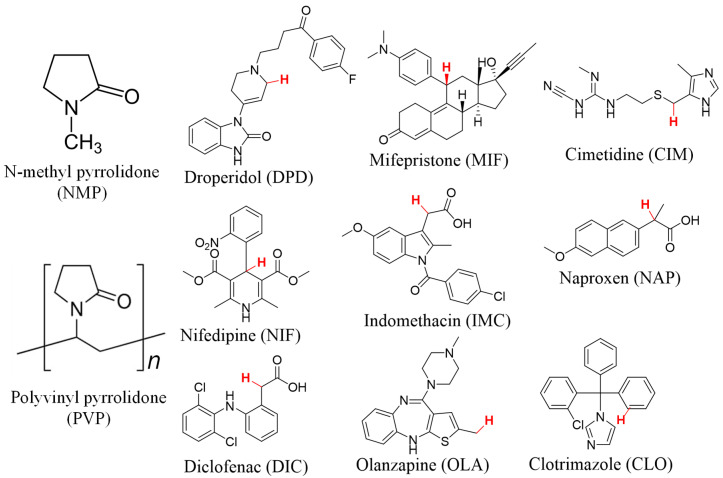
Chemical structure of NMP, PVP, and the selected drugs. Each drug’s H-atom with the lowest C–H BDE value is shown in red as reported in reference [32]. For PVP, *n* is the number of repeating monomer units.

N-methyl pyrrolidone (NMP) was procured from Sigma Aldrich (Steinheim, Germany). Five of the drug compounds namely, droperidol (DPD), mifepristone (MIF) cimetidine (CIM), naproxen (NAP), clotrimazole (CLO) were purchased from Merck (Sigma Aldrich^®^, Vienna, Austria), while indomethacin (IMC), nifedipine (NIF), and diclofenac (DIC) were obtained from TCI chemicals^®^ (Tokyo, Japan). Olanzapine (OLA) was purchased from Dr. Reddy’s laboratories (Hyderabad, India). All the selected drugs had a chemical purity above 99.90%, and their chemical structures are shown in Figure 1. Polyvinylpyrrolidone (PVP) K-60 was procured from Merck (Sigma Aldrich^®^, Vienna, Austria). The weighing of solid powders was done using a laboratory analytical balance. Ultrapure water was obtained from a TKA water purification unit (Vienna, Austria). Accelerated oxidation stability was conducted using a high temperature/pressure oxidation testing device, RapidOxy^®^ (Anton Paar GmbH^®^, Graz, Austria).

### 2.2. Methods

#### 2.2.1. Identification of Drugs by Differential Scanning Calorimetry (DSC)

Selected drug compounds were analyzed by DSC 204 F1 Phoenix (NETZSCH, Selb, Germany) to identify the melting temperatures as a confirmation that the received samples were crystalline. A reheating cycle of the quenched melt was also performed to determine the glass transition temperature (T_g_). The DSC was equipped with an autosampler and intra-cooler (to provide the controlled heating/cooling rate and temperatures). Approximately 5–10 mg of powder mass was placed in an aluminum pan, covered with a lid with a pinhole, and measured in heat–cool–heat mode. In the first heating cycle, samples were heated from 20 to 200 °C at a linear heating rate of 5 °C min^−1^. The samples were cooled back to 0 °C at a rate of 10 °C per min and reheated until 200 °C at the same initial heating rate of 5 °C per min to observe the T_g_ and other recrystallization events. Temperature calibrations were performed using indium. Following on from RapidOxy^®^ treatment the sample were analyzed by employing a modulated DSC method involving a first heating step from 20 °C to 250 °C with a heating rate of 5 °C min^−1^ with an amplitude of ±0.5 °C using a modulation period of 40 s. This test aimed to evaluate the T_g_ and inspect any changes in the solid-state properties.

#### 2.2.2. Identification of Drugs by Powder X-ray Diffraction (pXRD)

Powder X-ray diffraction (pXRD) experiments were performed with Bruker AXS-Siemens D5005 diffractometer (Dresden, Germany) equipped with 2.2 kW sealed Cu (IV) Kα wavelength 1.54060 source and NaI (Tl) scintillation counter detector to confirm that the solid state was crystalline. Samples were placed on a ϕ = 150 mm sample holder and scanned from 2θ values 3–40° using step size 0.04° every second in the reflection mode. The data were plotted using Origin software (OriginLab Corporation, Northampton, MA, USA).

#### 2.2.3. Solid State Stress Experiment Using RapidOxy^®^

Pre-milled PVP K-60 was used as the solid-state stressor to induce autoxidation in selected drugs by physically mixing 10 parts of pre-milled PVP K-60 and the drug in a ratio of 10:1. The physical mixture (PM) was placed in the glass sample holder and charged to RapidOxy^®^ (Anton Paar GmbH, Graz, Austria)—a high temperature, oxygen pressurized equipment [33]. The exposure conditions were set to 100 °C and 700 kPa of oxygen pressure (pO_2_) for 48 h. At the end of exposure, the sample was removed and immediately analyzed using appropriate chromatographic methods. Control experiments were conducted by using 700 kPa N_2_ pressures at 100 °C.

#### 2.2.4. Particle Size Distribution and Volume-Specific Surface Area

Particle size distribution was established to normalize the differences in the particle sizes of different selected drugs, which could play a role in solid-state degradation. Particle size distribution was analyzed using a wet dispersion technique on a HELOS laser diffraction particle-size analyzer (Sympatec GmbH, Königsbrunn, Germany). Water was used to disperse about 15 mg of powder drug with 0.1% Tween-80 as the surfactant. An exception was CIM, where we used toluene as the dispersion medium with 0.1% Tween-80 as the latter aided in better dispersity. The sample was vortexed for 60 s to disperse the particles evenly, and 10 µL of suspension was added to the cuvette containing about 50 mL dispersion medium. A magnetic bead stirrer was used to keep the dispersion medium dynamic, and the particle-size distribution was recorded for two independent samples. Depending on the size of monodispersed particles, the software automatically calculates the average volume-specific surface area (VSSA). The extent of % degradation in solid samples was normalized to this term, and a correlation was aimed with respective C–H BDEs.

#### 2.2.5. Kinetic Study Using NMP as the Reaction Medium in RapidOxy^®^

Stock solutions of DPD, MIF and, OLA were prepared by weighing approximately 50 mg of each drug in 5 mL of NMP. The drugs were dissolved by ultrasonication for 5 min generating a concentration of 10 mg mL^−1^ in NMP. A representative sample volume of 25 µL was removed from each stock solution and placed in DSC pans made from aluminum, (Netzsch Proteus, Selb, Germany) in triplicate. Therefore, each pan contains approximately 250 µg of the drug. These pans were placed in a petri dish and exposed to thermal-oxidative headspace using RapidOxy^®^. These samples were exposed to the temperatures of 45 °C, 50 °C, 55 °C, and 60 °C at an oxygen pressure of 700 kPa. Triplicate samples were withdrawn at 1 h, 2 h, 3 h, 4 h, 5 h, and 6 h intervals to generate the kinetics of autoxidation. For MIF, an additional time point at 12 h exposure was also investigated. As a control experiment, triplicate samples were exposed under nitrogen gas pressure (instead of oxygen) by keeping the same pressure, i.e., 700 kPa, for the last time point 6 h. Samples for UPLC analyses were generated by placing each DSC pan in an Eppendorf, and 1 mL of diluent comprising acetonitrile (ACN):water 70:30 was added. The sample was dissolved by manually shaking the closed Eppendorf for 10 s. Next, 800 µL of the dissolved sample was removed from Eppendorf and placed in a UPLC vial ready for UPLC. The injection volume in each method was kept at 0.5 µL, and the sample was analyzed using the reverse-phased gradient methods as specified in Table S1. Thus, the drug concentration of the injected sample is 125 µg mL^−1^. Degradation kinetics in the NMP solution was plotted, and the rate constants (k) (i.e., the slope) were obtained by using Microsoft Excel (version 2108, Washington, DC, USA). The respective data was used to generate Arrhenius plots, and the relevant Arrhenius parameters were derived.

#### 2.2.6. Ultra-Performance Liquid Chromatography (UPLC)

Chromatographic analysis was performed on Waters Acquity UPLC (Milford, MA, USA) using a photodiode array (PDA) detector. A stability-indicating method (SIM) was developed for each drug to separate the drug’s peak from those of the degradation products (DPs) by using different combinations of mobile phases and columns. The details are tabulated in Table S1. Relative quantification in the solution-state stress was performed by integrating the area under the peak (AUP) of the drug and DPs. The degradation can be calculated as shown in Equation (1).

% degradation = 100 − % AUP of drug(1)

Owing to the variations in degradation that could potentially result from the differences in absorption of DPs, an appropriate wavelength called as the ‘isosbestic wavelength’ was used. The isosbestic wavelength is the wavelength where the absorbances of species (DPs and drugs) become identical, thereby eliminating any variations associated with their response in the observed signal (chromatogram) [34]. For solid-state experiments, the extent of degradation at the end of exposure was calculated by using Equation (1). Absolute quantification of the drug (remaining) was not attempted as the emphasis was to compare the rates or propensities of drug degradation with time. The spectral purity of the eluting peaks was evaluated by using the ‘peak purity’ function in the photodiode array processing channel in Empower-3 software. The methods were considered as fit for purpose based on the spectral homogeneity across the apex and baseline of the peaks after normalizing for the baseline noise and solvent effects.

#### 2.2.7. Liquid Chromatography-Mass Spectrometry (LC-MS)

Selected samples of DPD, CIM, and MIF exposed with PVP in RapidOxy^®^ were analyzed using LC-MS to confirm the reaction mechanism under high oxygen pressure conditions. Samples were run on a Waters Synapt G2-Si (Macclesfield, UK) operating in positive ion electrospray mode connected to UPLC (Waters, Acquity, Milford, MA, USA) with a quaternary pump. The capillary voltage was kept at 3 kV while the cone voltage was 30 V with a gas flow of 50 L/h. The source temperature was set to 150 °C while the desolvation gas temperature was 600 °C. The desolvation gas flow was kept at 1200 L/h. The acquisition range in MSe mode was kept between 100–1200 m/z. The detailed structure elucidation of the DPs was out of the scope of this work, and only the obtained line spectra with molecular ion peaks and errors were used to elucidate the most plausible structure.

## 3. Results

### 3.1. Identification of Selected Compounds by DSC and pXRD

Eight compounds were selected based on a variation in their calculated C–H BDEs ranging from 70 kcal mol^−1^ to 85 kcal mol^−1^ (Table 1). The chemical structure of the selected compounds and their abstractable hydrogen is shown in Figure 1. Clotrimazole (CLO) was chosen as the ninth compound for use as a negative control, since it possesses the highest BDE C–H with no reports of autoxidation.

**Figure 2:**
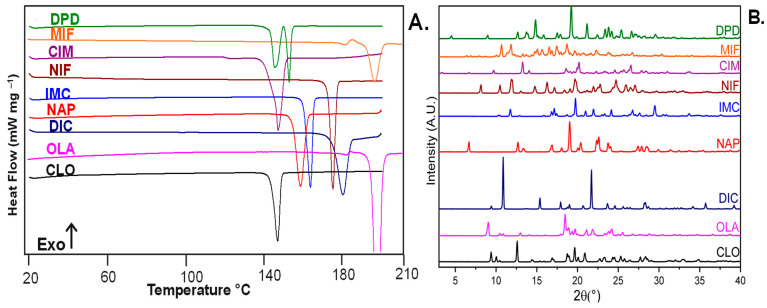
DSC heating curves (**A**) and pXRD profiles (**B**) of selected drug compounds.

All the received materials were crystalline, as evidenced by a melting event in the first heating run of the DSC scan. Experimental T_g_ values are shown in the second heating run of DSC results (see Supplement Figure S1). The pXRD patterns of selected compounds (Figure 2B) revealed characteristic Bragg peaks indicating the crystalline nature of the received samples.

One can see from Figure 2A that three of the received samples are metastable polymorphs of DPD, MIF, and OLA, as they have two melting events. It has been reported that the metastable polymorph of DPD has a melting range of 139.8–148.5 °C while the stable polymorph has a melting range of 146.5–148.5 °C [44]. A report is available for MIF, which is consistent with our findings that the sample is a metastable crystal (Form D) [45]. Comparing DSC and pXRD profiles for CIM indicated that the received sample is metastable Form C [46]. Likewise, reports are consistent with the received OLA sample being Form II [47,48]. The rest of the compounds are crystalline, stable polymorphs.

### 3.2. Solid State Degradation Using RapidOxy^®^

The exposure of PM comprising pre-milled PVP K-60 containing free radicals in combination with DPD (10:1) in RapidOxy^®^ under high-pressure O_2_ (700 kPa) at elevated temperature (100 °C) was reported to produce autoxidation [28]. Hence, we employed the same protocol and exposed the selected samples under RapidOxy^®^ conditions. Figure S2 (see Supplementary Materials) captures details of chromatograms of samples exposed in RapidOxy^®^ under 700 kPa for 48 h at 100 °C. The extent of degradation of drugs is tabulated in Table 2.

**Figure 3:**
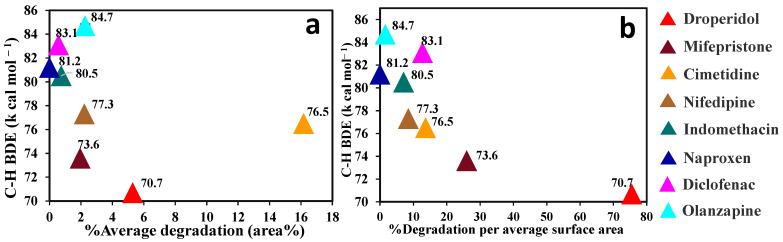
Comparison of C–H BDE versus solid-state degradation of selected compounds without (**a**) and with surface area normalization (**b**). The numbers next to the symbols are the calculated C–H BDE of the respective compounds.

Figure 3a compares the extent of solid drug degradation with their C–H BDEs. A correlation exists in most of the compounds, except CIM and OLA. Interestingly, NIF, which is known to undergo oxidation [49], gives a better correlation than CIM, although both these compounds have similar C–H BDEs.

To further investigate the impact of particle surface area on the observed correlation, the degradation values were normalized against volume-specific surface area (VSSA).

It can be observed in Table 2 that OLA has the lowest D_50_ and D_10_ values and the highest VSSA among the selected compounds. As shown in Figure 3b, OLA and CIM show an improvement in the expected correlation after normalizing their surface area. The Pearson’s correlation coefficient (R) of the extent of degradation of these eight compounds with respect to their C–H BDE is −0.8052, and the *p*-value is 0.0159, indicating a slightly improved correlation score and a statistically significant result.

These findings suggest that autoxidation of drugs in solid state shows clearer trends with respect to their C–H BDE values (that were previously calculated for the unionized molecular species) upon the particle size/surface area normalization of solids. This is an encouraging finding as our precedent study reported that autoxidation kinetics and energetics of drugs in solution tend to show a poor correlation with their calculated C–H BDEs [32]. A parallel reaction under nitrogen pressure was performed to account for the contribution of pressure and temperature on the anaerobic reaction. There was negligible degradation when this reaction was performed in RapidOxy^®^ under equivalent nitrogen pressures and temperature instead of oxygen pressures (see Supplement Figure S3). Thus, the autoxidation of drugs in the presence of pre-milled PVP K-60 depends on the presence of free radicals as well as oxygen. The reaction does not occur only due to the elevated temperatures and pressure.

### 3.3. Autoxidation Kinetics of Selected Drugs in NMP

**Figure 4:**
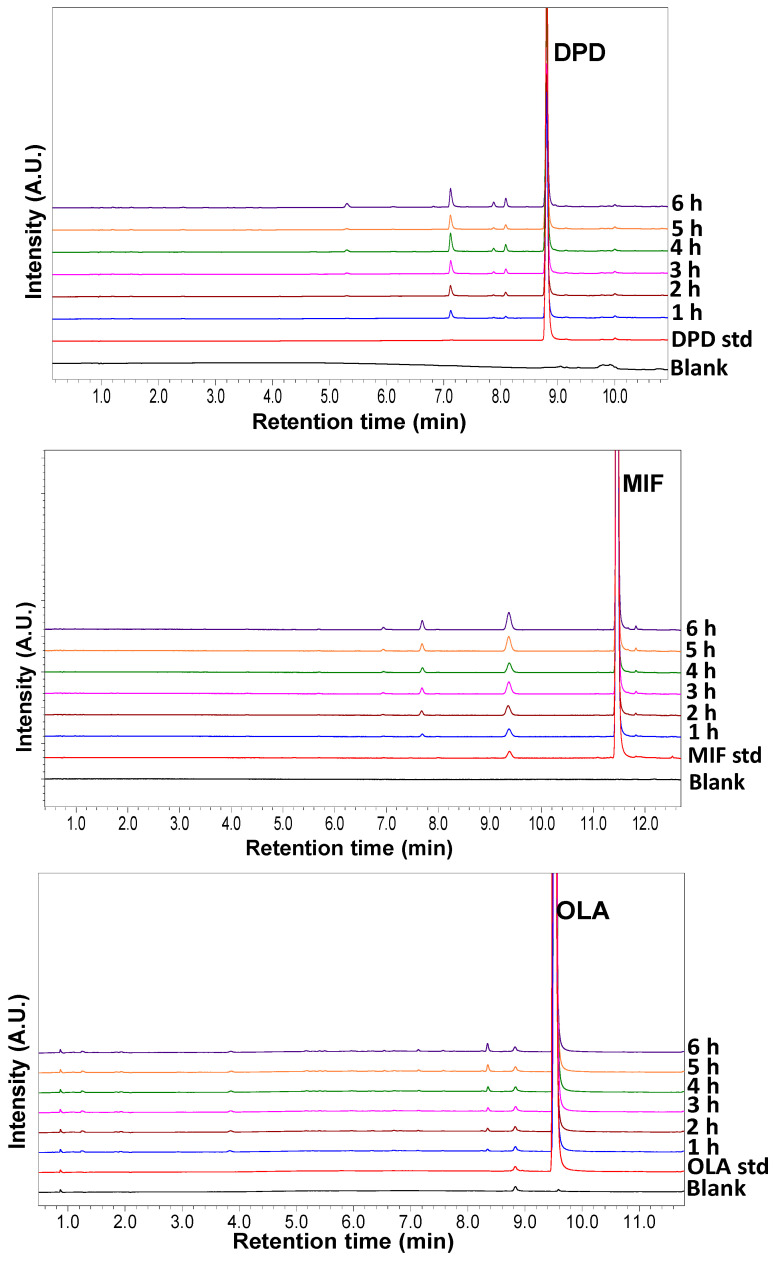
Overlaid zoomed chromatograms depicting enrichment of autoxidative DPs from drugs when dissolved in NMP and exposed in RapidOxy^®^. No additional peaks were observed in the chromatogram beyond the displayed retention time range. Key: std-drug standard (time zero sample), 1–6 h are the selected kinetic time points.

In this work, the expected role of NMP was to serve both as a solvent to dissolve the drugs, and as a reactant to generate free radicals and initiate autoxidation. While no degradation was observed for the three selected drugs (DPD, MIF, and OLA) at time zero, it is expected that NMP undergoes autoxidation when exposed in RapidOxy^®^ at elevated temperatures above 45 °C. The oxidation of NMP could have generated hydroperoxides that autoxidize the drugs similar to that observed in the solid state (where pre-milled PVP K-60 is used as the excipient). Appropriate chromatograms showing the autoxidation kinetics of these three drugs exposed at 60 °C/700 kPa O_2_ pressure are shown in Figure 4.

**Figure 5:**
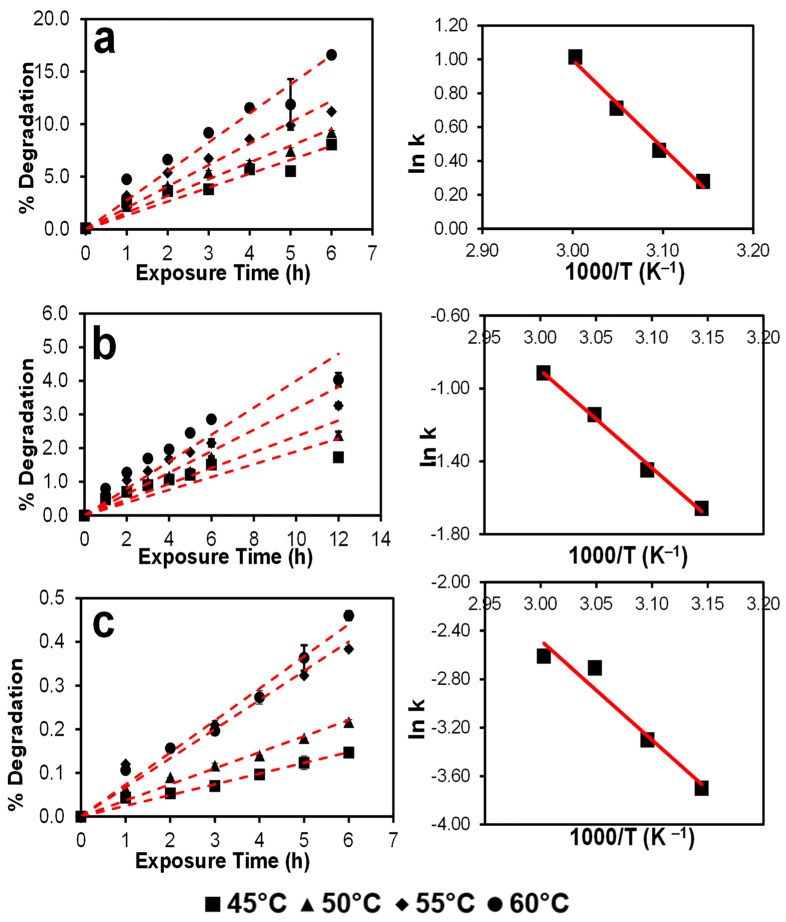
Plots depicting autoxidation kinetics (on the right side) of DPD (**a**), MIF (**b**), and OLA (**c**) in NMP exposed in RapidOxy^®^ conditions and their respective Arrhenius plots (on the right). The key for left side plots: the symbols represent the average extent of degradation at respective temperatures, and the error bars indicate standard deviation (n = 3 samples), while the red dotted line represents a linear fit. The R^2^ was above 0.98 in all the cases. A linear fit was best approximated in the Arrhenius plots on the right (R^2^ was above 0.95).

As evident in Figure 5, a linear degradation kinetic is observed for all three drugs. This trend enabled the application of a linear fit to the experimental data points and extraction of the reaction rates (slopes). It can also be speculated that autoxidation follows a first-order degradation kinetics in the solution state.

DPD shows both a much faster and greater extent of autoxidation, followed by MIF. The slowest degradation kinetics was evidenced for OLA. While it is important to mention here that a host of different minor DPs were formed from OLA, only the DP at RRT 0.877 was found to enrich over the exposed periods. Hence, this peak has been integrated (considered as the main oxidation product). The degradation values of MIF are very similar at all the four selected temperatures until the first 6 h of exposure; and a 12 h timepoint was thus included. An interesting trend is observed for OLA, where the fitted (dotted) lines are very similar for 45 and 50 °C. However, abruptly higher degradation kinetics are observed at 55 and 60 °C, indicating the temperature dependence of autoxidation for OLA. Visualization of the Arrhenius plots on the right side of these plots demonstrates a reasonably linear relationship between the inverse of exposed temperature and the natural logarithm of degradation rates.

The derived Arrhenius parameters, i.e., activation energy (E_a_) and the pre-exponential factor (A) for three drugs in NMP, are shown in Table 3. As can be seen, the activation energy barriers increase when moving from DPD to OLA, which is to be expected given that DPD undergoes autoxidation more readily than OLA. Also noteworthy is the incremental trend in the pre-exponential factor of these drugs.

**Figure 6:**
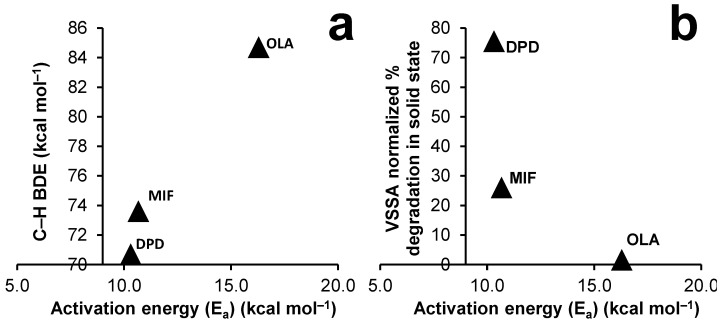
Plots demonstrating (**a**) the relationship between the E_a_ of drug autoxidation in NMP solution (RapidOxy^®^ stress) and their C–H BDE and (**b**) a comparison between E_a_ of drugs autoxidation in NMP solution and volume-specific surface area normalized degradation extent in the solid state.

Alongside the increasing values of autoxidation rates and E_a_, it is noteworthy that there is also a rising trend in the C–H BDEs, as shown in Figure 6a. A distinct positive relationship between the thermodynamic factor (C–H BDE) and the kinetic factor (E_a_) suggests that C–H BDE indeed may be a good descriptor to probe the autoxidation relationship, provided that there is no inert solvent present. Also, the same figure (Figure 6b) compares E_a_ obtained in NMP solution stress to the surface area normalized autoxidation in the solid state. It is plausible from Figure 6b that an inversely exponential relation may exist between the E_a_ of drugs (obtained in the NMP) to the degradation obtained in solid state in the presence of PVP. It can be also proposed from this figure that drugs showing an E_a_ above 15 kcal mol^−1^ in the solution stress may not show any notable degradation in the solid state. However, verifying this relationship would require appropriate oxidative studies to be conducted in a significant number of drugs with distinct chemical groups and is acknowledged to be a limitation of the present study.

## 4. Discussion

The utility of C–H BDE in assessing the autoxidation propensities of drugs is evidenced in the literature [50]. This study attempted to evaluate the autoxidation of eight selected drug compounds ranging in the C–H BDE from 70–85 kcal mol^−1^ in the solid state. An extensive solution state autoxidation was previously performed and published by our lab. Also, one drug was chosen as a control, i.e., CLO having a C–H BDE of 107 kcal mol^−1^. Thermal and X-ray diffraction experiments revealed the crystalline nature of these received drugs. Observation of the glass-forming potential by heat–cool–reheat cycle in DSC revealed a vast difference in the T_g_ values with a median range of 40–60 °C. Upon exposure at RapidOxy^®^, it can thus be inferred that the small fraction (undetectable by DSC) of the amorphous phase, if present initially or formed during degradation, would exist in the supercooled liquid state during the solid-state stress study at 100 °C. The single exception to this was for MIF where a T_g_ was observed at 105 °C which is 5 °C above the exposure temperature used in RapidOxy^®^. No clear relationship was found, however, between the differences in temperature of exposure/physical state 100 − T_g_ and the extent of degradation.

The application of high oxygen pressure and a high temperature in combination with a radical-enriched PVP excipient was reported to induce autoxidation of DPD in our previous study [28]. The DPs formed in this case (solid state) were the same as those formed under a solution stress study conducted in AIBN suggesting that these are autoxidative. The results from the RapidOxy^®^ treatment of several of the selected solid drug compounds in the physical mixture with pre-milled PVP K-60 indicate the formation of autoxidative DPs (see Supplement Figure S2). Control experiments conducted in parallel involved exposing the drug pre-milled PVP K-60 physical mixture to nitrogen gas pressures (700 kPa) at the same temperature. This study did not evidence a formation of oxidative DPs, suggesting that oxygen is a prerequisite for inducing the degradation (data shown in Supplement Figure S3) and high temperature with a free-radical enriched excipient source is not able to induce autoxidation. No degradation was observed for DIC, NAP, and CLO until the study period pointing to the critical challenge of oxidizing these solid drugs. A possible reason for the remarkable oxidative stability of DIC and NAP could be attributed to their high C–H BDE apart from their ionic character. The latter makes the abstraction of an H-atom by peroxy-radical challenging. A point of interest was that a much higher degradation was observed for CIM (approximately 13% by relative area %). Normalizing the extent of degradation with a volume-specific surface area improved the drugs’ autoxidation trend with respect to C–H BDE. However, a perfect correlation was not achieved. This may be ascribed to the difference in the amorphization/glass-forming potential of each of these compounds, steric factors governing the accessibility of molecular oxygen to the reactive site in the molecular crystal lattice, and the diversity of chemistry or functional groups involved in the reaction.

Technical limitations of this work can be enumerated as the following. First, BDE is a gas-phase descriptor, and an experimental determination of this value may not be feasible under standard laboratory conditions for solid state drugs. Second, the BDE can be a potentially labile site in the drug molecule where several autoxidation mechanisms may mediate due to hydrogen abstraction. This is evident in the case of MIF, where the majority of formed DPs are characterized as demethylated and di-demethylated products instead of a product resulting from C–H abstraction alone. On a positive note, there is evidence of the formation of a dehydrogenated product in DPD, which aligns with our expectation, although minor DPs, one of which is presumed to be N-oxide of DPD, are also formed (data shown in Supplement Figure S4). Likewise, in the case of OLA, the structure of formed DP is consistent with the N-oxide product, where a direct addition of 16 m/z is evidenced.

Overall, the relationship of the calculated C–H BDE with the (surface area normalized) extent of autoxidation in solid state is more apparent here than that with the autoxidation of the same set of drugs in aqueous AIBN solution that we found previously [32]. For the latter case, we found experimentally that the ionized state plays a crucial role (pH−pKa); thus, the calculation of C–H BDE of ionized species might be necessary. In the present case of solid-state autoxidation, it is tempting to assume that most of the reacting drug molecules will be present in a unionized state. Therefore, the C–H BDE value calculated for the neutral species will still show a reasonable relation to the extent of autoxidation. The value of micro-environmental pH measured of drug–PVP mixture with respect to the respective drug pKa showed no apparent association to the extent of autoxidation, pointing to the trivial to no importance of the ionization state (see Supplement Figure S5). One of the main limitations of the solid-state degradation study of this work is that the temperature used is much higher than that in the conventional accelerated and long-term conditions, and also above the T_g_ of many drugs. Therefore, the relative rates at real-time storage cannot be inferred from the present solid-state data. Further study involving multiple drug-to-PVP ratios, temperatures below and above T_g_, and single time points (kinetics) is necessary and can be performed using RapidOxy^®^. Further, the effect of oxygen pressure on such solid-state reactions needs to be investigated and compared to the ambient pressure.

While the oxidation of crystalline drugs with pre-milled PVP K-60 in RapidOxy^®^ can be slower/limited for several reasons, it must be acknowledged that it provides a more realistic reference to the autoxidation risk involved in a typical formulation. Hence, in this study, conducting the drug’s autoxidation in NMP (monomer of PVP) as a surrogate stressor was technically evaluated, in particular to speed up the reaction and to erase the impact of powder properties. Experiments conducted by dissolving drugs in NMP (for DPD, MIF, and OLA), and exposing them in RapidOxy^®^ enriched the formation of the same DPs as those that were formed in the drug–pre-milled PVP physical mixture. Notably, higher drug degradation was observed here, even at much lower temperatures, enabling the exploration of the autoxidation kinetics of these drugs. Applying a linear model fit (y = mx + c) to the degradation data indicated that the reaction would follow a first-order oxidation. Also, the value of the slope derived was considered as the reaction rate, and an Arrhenius plot were constructed for the three drugs. An incremental trend between the derived activation energy values and the C–H BDE suggested a positive relationship. Likewise, a declining trend was noted among the normalized degradation extent (in the solid state) and the derived activation energy in the NMP, suggesting a good relationship between the solid state and solution state autoxidation when PVP monomer analog (NMP) was used. It can be speculated from Figure 6b that for drugs with activation energy around 10 kcal mol^−1^, a greater extent of autoxidation in the solid state can be expected, whereas above 15 kcal mol^−1^ the autoxidation in solid state becomes negligible. While it is acknowledged that future experiments should be targeted to explore the autoxidation relationship to C–H BDE by using multiple drug compounds with differing chemistries, it is noteworthy here that the use of NMP (as a PVP-surrogate) can be a useful screening tool to study autoxidation propensities of drugs in solid formulations. Moreover, as the use of a high boiling point (202 °C) NMP avoids the need of any aqueous and/or organic solvent, the impact of ionization and solvent evaporation on the autoxidation results will be minimal.

## 5. Conclusions

In the present study, the autoxidation propensity of a diverse set of drugs was investigated in solid and solution states by the application of an elevated temperature and pressurized oxygen setup (RapidOxy^®^). A positive relationship between the surface area-normalized autoxidation extent of drugs was observed with their calculated C–H BDE values, suggesting it to be a useful descriptor for studying the autoxidation propensities of drugs in the solid state. While the autoxidation kinetics of solid drugs in the physical blend with PVP excipient may be complicated by several factors (particle size, surface area, moisture, and amorphous contents, etc.), it is feasible that a faster indication of autoxidation propensity could be discerned by conducting a surrogate experiment in the solution state by using NMP (monomer of PVP). A positive relationship was evidenced between the activation energies (E_a_) of three selected drugs autoxidized in NMP to their calculated C–H BDE suggesting a possible prediction of autoxidation behavior; however, the relationship between the E_a_ and autoxidation propensity of solid drugs would need a thorough verification for a diverse number of drugs. The present work presents a novel utility of NMP in a high oxygen pressurized setup as a forced autoxidation tool that can act as a surrogate to screen the autoxidation potential of drugs in solid formulation, and especially for those containing PVP as excipient.

## Figures and Tables

**Table 1:** List of selected compounds for solid- and solution-states autoxidation study and their properties.

	Experimental Values			Theoretical/Calculated Values		
Drug	Melting Point T_m_ (°C) Onset	Melting Enthalpy (J g^−1^)	Glass Transition Temperature (T_g_) (°C) Onset	C–H BDE (kcal mol^−1^)	pKa	Reference for pKa
Droperidol (DPD)	151	103.14	28.90	70.70	7.46	[35]
Mifepristone (MIF)	192	68.27	105.30	73.60	4.89	[36]
Cimetidine (CIM)	144	142.50	43.70	76.50	6.80	[37]
Nifedipine (NIF)	172	114.80	40.70	77.30	3.93	[38]
Indomethacin (IMC)	159	105.40	40.60	80.50	4.50	[39]
Naproxen (NAP)	154	123.00	*	81.20	4.15	[40]
Diclofenac (DIC)	175	131.40	7.20	83.10	4.15	[41]
Olanzapine (OLA)	194	135.40	66.60	84.70	7.80	[42]
Clotrimazole (CLO)	143	84.78	27.90	107.60	4.10	[43]

*: Naproxen recrystallized during the cooling run in DSC hence, T_g_ could not be observed.

**Table 2:** Extent of drug degradation in RapidOxy^®^ and their particle size distribution, and average volume-specific surface area of selected drugs.

Drug	Area%	Range	D_50_ (µm)	D_10_ (µm)	Volume-Specific Surface Area (VSSA) (n = 2)		VSSA Normalized
	Degradation	n = 2			Average (m^2^/cm^3^)	Range	Average Degradation%
DPD	5.29	0.03	171.82	82.32	0.07	0.03	75.50
MIF	1.95	0.07	111.31	42.14	0.08	0.01	26.00
CIM	16.17	0.07	17.52	2.34	1.18	0.14	13.70
NIF	2.22	0.01	27.25	9.45	0.26	0.01	8.52
IMC	0.75	0.03	106.57	33.23	0.11	0.01	7.10
NAP	0.37	0.06	42.10	18.85	0.18	0.03	0.00
DIC	0.58	0.21	168.82	76.42	0.05	0.01	12.78
OLA	2.26	0.38	13.44	2.40	1.44	0.04	1.57
CLO	0.00	–	37.12	17.54	0.21	0.04	0.00

**Table 3:** Experimental autoxidation rates and Arrhenius parameters for the selected drugs in NMP solution stress.

Drug	Temperature (°C)	Autoxidation Rate k (h^−1^)	Temperature (K)	1000/T (K^−1^)	ln k	Slope	E_a_ (kcal mol^−1^)	ln A	A
DPD	45	1.320	318	3.145	0.278	−5.192	10.316	16.570	1.571 × 10^7^
	50	1.588	323	3.096	0.463				
	55	2.035	328	3.049	0.710				
	60	2.756	333	3.003	1.014				
MIF	45	0.190	318	3.145	−1.660	−5.371	10.672	15.213	4.05 × 10^6^
	50	0.235	323	3.096	−1.448				
	55	0.318	328	3.049	−1.144				
	60	0.400	333	3.003	−0.915				
OLA	45	0.025	318	3.145	−3.701	−8.198	16.290	22.113	4.014 × 10^9^
	50	0.037	323	3.096	−3.302				
	55	0.067	328	3.049	−2.708				
	60	0.073	333	3.003	−2.612				

## Data Availability

The supporting data (with raw data) is available as Supplement file.

## Supplementary Materials

The following supporting information can be downloaded at: https://www.mdpi.com/article/10.3390/pharmaceutics15030848/s1, Table S1: Chromatographic method conditions used in solid state autoxidation stress (RapidOxy^®^). Figure S1: Experimental glass transition (onset) temperatures (T_g_) of selected compounds obtained in the 2nd heating scan by heat-cool-reheat cycle in DSC. Inset for DIC shows magnified T_g_ region. NAP sample recrystallized in the cooling run, thus is not reported here. Figure S2: Overlaid chromatograms of solid-state oxidation stress on the selected drug candidates with pre-milled PVP K–60 (Roxy indicates RapidOxy^®^). Figure S3: Control experiments under elevated nitrogen pressures (in RapidOxy^®^) and solution stress experiment using H_2_O_2_. (std–standard; PM–physical mixture; ROXY–RapidOxy). Figure S4: Structures of DPs formed in Drug:PVP PM (1:10) under RapidOxy^®^ conditions as characterized by LC–MS analyses. Figure S5: Comparison of extent of ionization (pH–pKa) and the VSSA normalized degradation in the solid state drug:premilled PVP K–60 (1:10) mixture before (A), and after RapidOxy^®^ exposures (B). * PM stands for Physical mixture and VSSA is the volume specific surface area.
