# Supplementary material for: Screening Autoxidation Propensities of Drugs in the Solid-State Using PVP and in the Solution State Using N-Methyl Pyrrolidone
Source: Pharmaceutics. 2023 Mar 5;15(3):848. doi: 10.3390/pharmaceutics15030848 (PMC10058359; doi:10.3390/pharmaceutics15030848)
Supplement: Supplementary file 1 [file pharmaceutics-15-00848-s001.zip › pharmaceutics-2238866-supplementary.pdf]

Supplementary Materials: Screening autoxidation propensities of drugs in the solid state using PVP and in the solution state using N-methyl pyrrolidone

Jayant Iyer[1], Anjali Karn[1], Michael Brunsteiner[1], Andrew Ray[3], Adrian Davis[4], Isha Saraf[1] and Amrit Paudel[1,2*]

Table S1. Chromatographic method conditions used in solid state autoxidation stress (RapidOxy®).

Drug	Column	Mobile Phase	Method details (Time in minutes/%B organic)	Flow rate (ml/min)	UV λmax (nm)	Injection volume (µL)	Column temperature (°C)	Autosampler temperature (°C)	Concentration of stress solution (µg mL−1)
DPD	WATERS ACQUITY UPLC BEH C–8 (2.1mm*100mm) 1.7µ	A: 10 mM AA and B: MeOH (100% v/v) pH 5.05	T_{min}/B%: $T_{0.0}$/25; $T_{2.0}$/25; $T_{10.0}$/75; $T_{12.0}$/90; $T_{13.0}$/25; $T_{15.0}$/25	0.350	235	1	40	25	500
MIF	WATERS ACQUITY UPLC HSS T3 (2.1mm*100mm) 1.8µ	A: 10 mM AF and B: ACN:MeOH 90:10 (100% v/v) pH 3.70	T_{min}/B%: $T_{0.0}$/25; $T_{1.0}$/25; $T_{5.5}$/40; $T_{8.5}$/40; $T_{11.0}$/80; $T_{12.0}$/80; $T_{12.10}$/25; $T_{15.0}$/25	0.350	305	0.5	30	10	500
CIM	WATERS ACQUITY UPLC BEH C–8 (2.1mm*100mm) 1.7µ	A: 10 mM AA and B: ACN (100% v/v) pH 9.02	T_{min}/B%: $T_{0.0}$/5; $T_{2.0}$/5; $T_{6.0}$/10; $T_{10.0}$/35; $T_{12.00}$/5; $T_{14.0}$/5	0.250	225	0.5	30	25	500
NIF	Agilent Poroshell eC–8 (100mm*3mm),2.7 u	A: 10 mM AF and B: MeOH (100% v/v) pH 4.1	T_{min}/B%: $T_{0.0}$/20; $T_{8.0}$/50; $T_{15.0}$/90; T_{16}/20; T_{18}/20	0.400	235	1	40	15	250
IMC	WATERS ACQUITY UPLC HSS T3 (2.1mm*100mm) 1.8µ	A: 10 mM AF and B: ACN (100% v/v) pH 3.1	T_{min}/B%: $T_{0.0}$/25; $T_{1.0}$/25; $T_{8.0}$/60; $T_{9.0}$/70; $T_{12.00}$/85; $T_{13.00}$/85; $T_{13.01}$/25; $T_{15.0}$/25	0.325	230	1	30	20	250
NAP	WATERS ACQUITY UPLC HSS T3	A: 10 mM AF and B: ACN (100% v/v) pH 3.1	T_{min}/B%: $T_{0.0}$/25; $T_{1.0}$/25; $T_{8.0}$/60; $T_{9.0}$/70; $T_{12.00}$/85; $T_{13.00}$/85; $T_{13.01}$/25; $T_{15.0}$/25	0.325	235	0.5	30	20	50

	(2.1mm*100mm) 1.8µ								
DIC	WATERS ACQUITY UPLC HSS T3 (2.1mm*100mm) 1.8µ	A: 10 mM AF and B: ACN (100% v/v) pH 3.1	T_{min}/B%: $T_{0.0}$/25; $T_{1.0}$/25; $T_{8.0}$/60; $T_{9.0}$/70; $T_{12.00}$/85; $T_{13.00}$/85; $T_{13.01}$/25; $T_{15.0}$/25	0.325	276	1	30	20	250
OLA	WATERS ACQUITY UPLC BEH C–18 (2.1mm*100mm) 1.7µ	A: 10 mM AA and B:ACN:MeOH(90:10% v/v) pH 5.61,	T_{min}/B%: $T_{0.0}$/10; $T_{2.0}$/10; $T_{10.0}$/40; $T_{14.0}$/80; $T_{14.10}$/10; $T_{17.00}$/10	0.300	250	0.5	30	10	500
CLO	WATERS ACQUITY UPLC BEH C–8 (2.1mm*100mm) 1.7µ	A: 10 mM AA and B: ACN (100% v/v) pH 9.02	T_{min}/B%: $T_{0.0}$/20; $T_{2.0}$/20; $T_{6.0}$/50; $T_{10.0}$/80; $T_{12.00}$/90; $T_{12.01}$/20; T_{15}/20	0.350	225	1	40	25	500

Abbreviations: AF–Ammonium Formate, AA–Ammonium Acetate, MeOH–Methanol, ACN–Acetonitrile

pharmaceutics

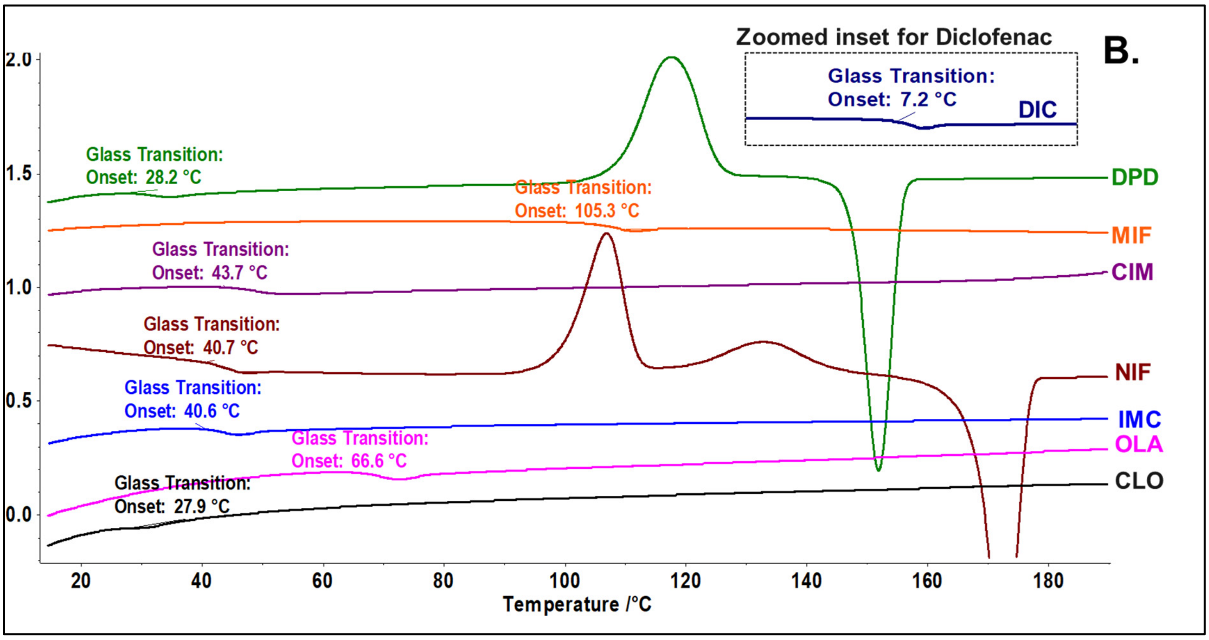

Figure S1. Experimental glass transition (onset) temperatures (T_g) of selected compounds obtained in the 2nd heating scan by heat–cool–reheat cycle in DSC. Inset for DIC shows magnified T_g region. NAP sample recrystallized in the cooling run, thus is not reported here. (Exotherm points upwards)

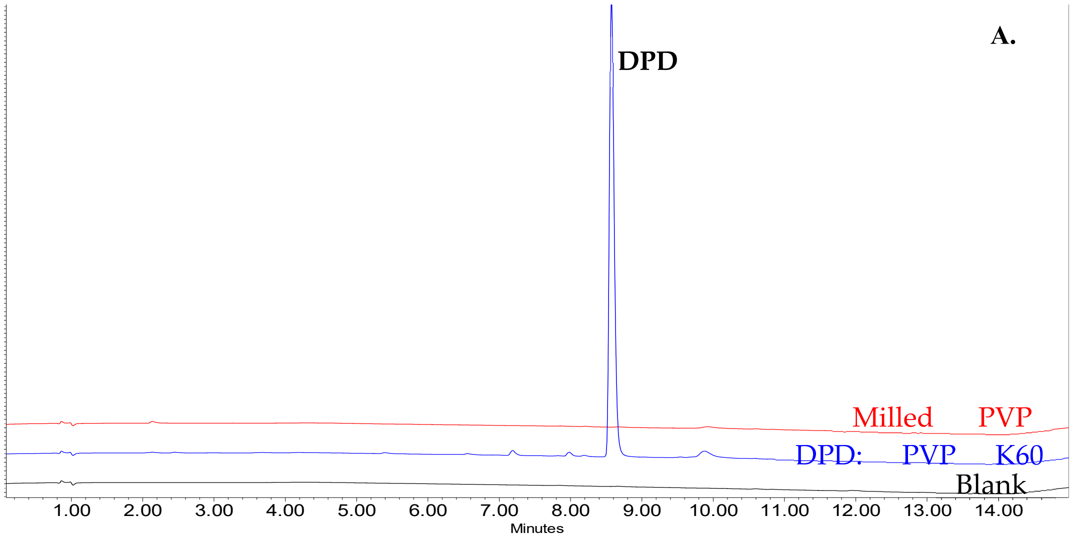
A.
DPD
Milled PVP
DPD: PVP K60
Blank
1.00 2.00 3.00 4.00 5.00 6.00 7.00 8.00 9.00 10.00 11.00 12.00 13.00 14.00
Minutes

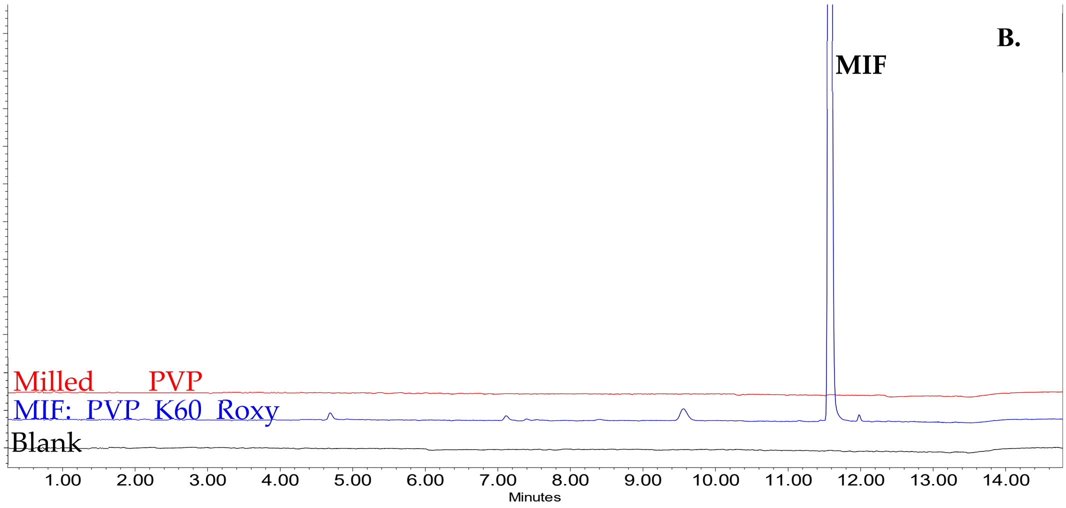
B.
MIF
Milled PVP
MIF: PVP K60 Roxy
Blank
1.00 2.00 3.00 4.00 5.00 6.00 7.00 8.00 9.00 10.00 11.00 12.00 13.00 14.00
Minutes

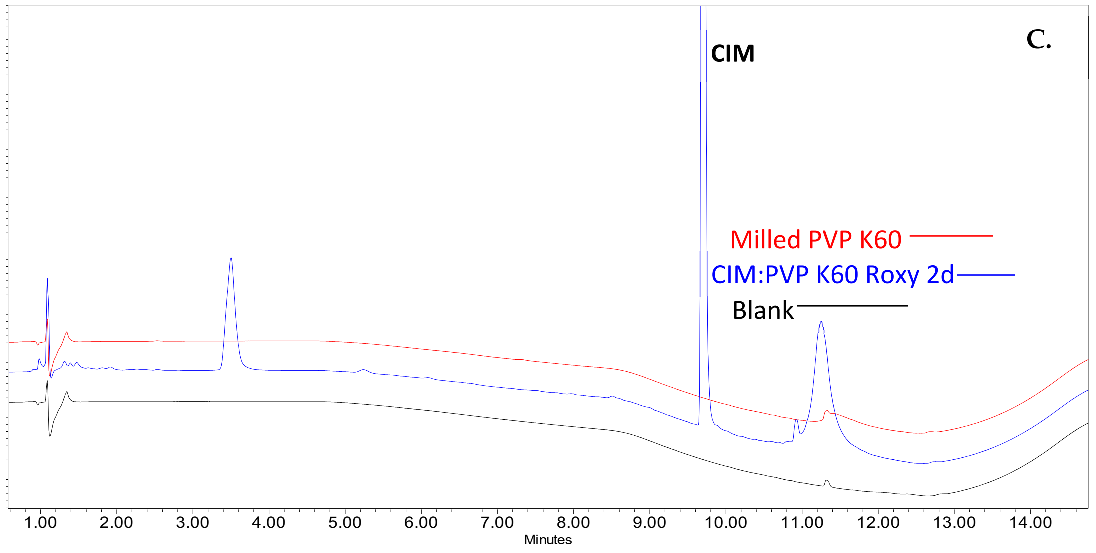
CIM
C.
Milled PVP K60
CIM:PVP K60 Roxy 2d
Blank
1.00
2.00
3.00
4.00
5.00
6.00
7.00
8.00
9.00
10.00
11.00
12.00
13.00
14.00
Minutes

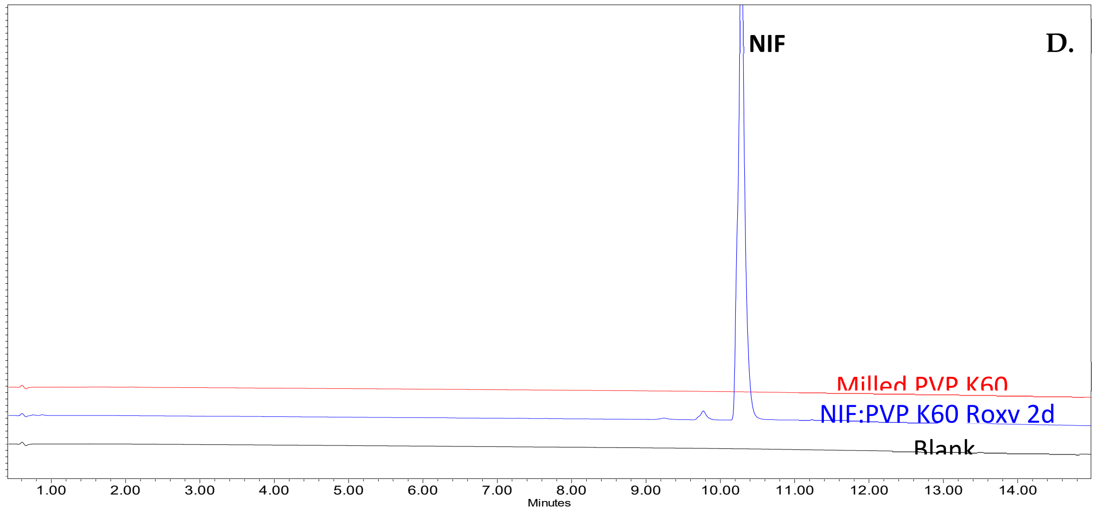
NIF
D.
Milled PVP K60
NIF:PVP K60 Roxy 2d
Blank
1.00
2.00
3.00
4.00
5.00
6.00
7.00
8.00
9.00
10.00
11.00
12.00
13.00
14.00
Minutes

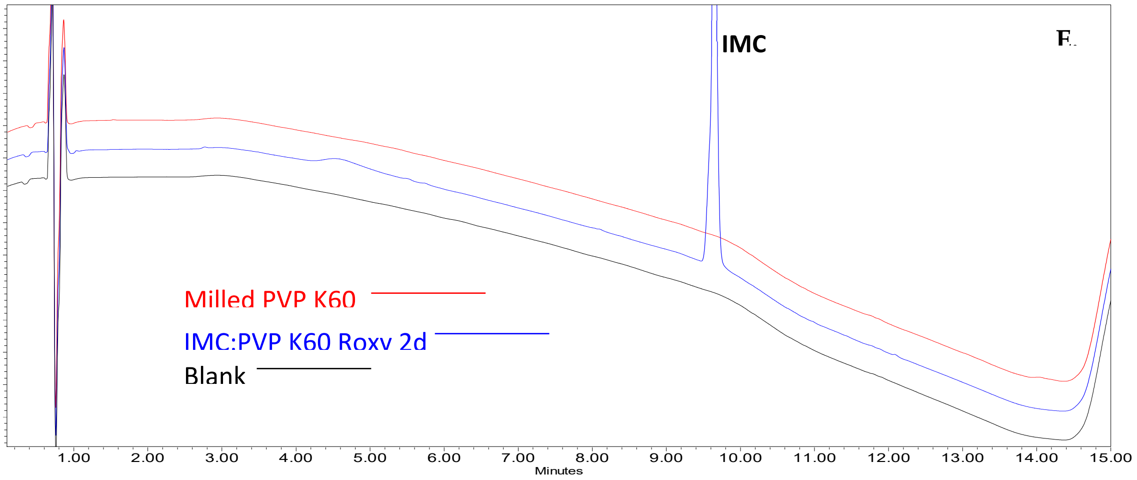
IMC
Milled PVP K60
IMC:PVP K60 Roxy 2d
Blank
1.00
2.00
3.00
4.00
5.00
6.00
7.00
8.00
9.00
10.00
11.00
12.00
13.00
14.00
15.00
Minutes

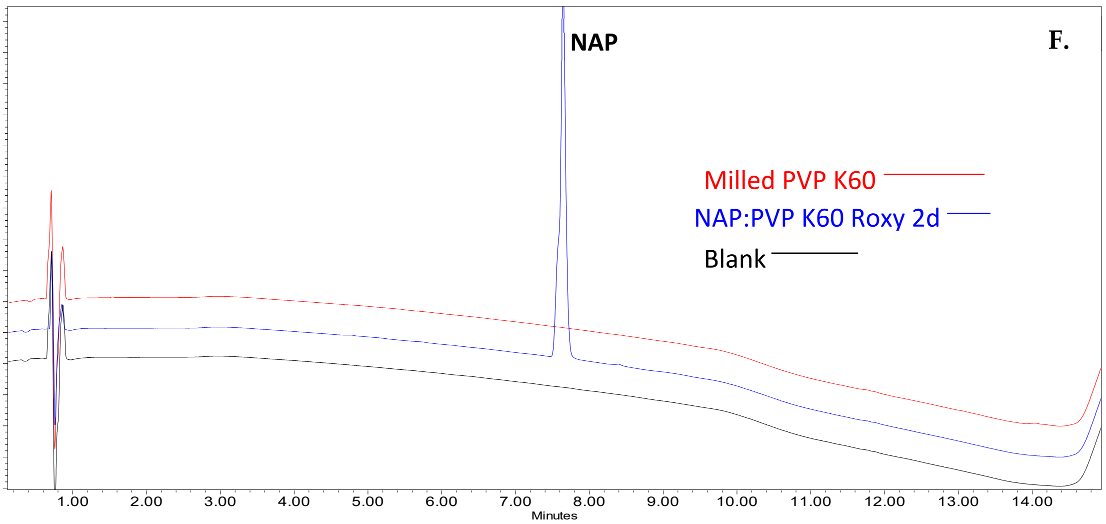
NAP
F.
Milled PVP K60
NAP:PVP K60 Roxy 2d
Blank
1.00 2.00 3.00 4.00 5.00 6.00 7.00 8.00 9.00 10.00 11.00 12.00 13.00 14.00
Minutes

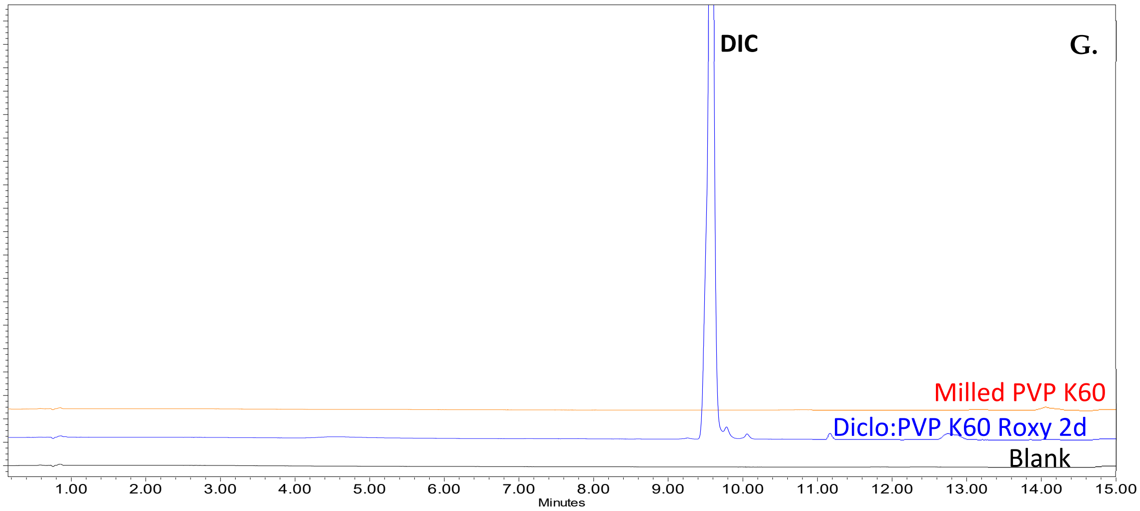
DIC
G.
Milled PVP K60
Diclo:PVP K60 Roxy 2d
Blank
1.00 2.00 3.00 4.00 5.00 6.00 7.00 8.00 9.00 10.00 11.00 12.00 13.00 14.00 15.00
Minutes

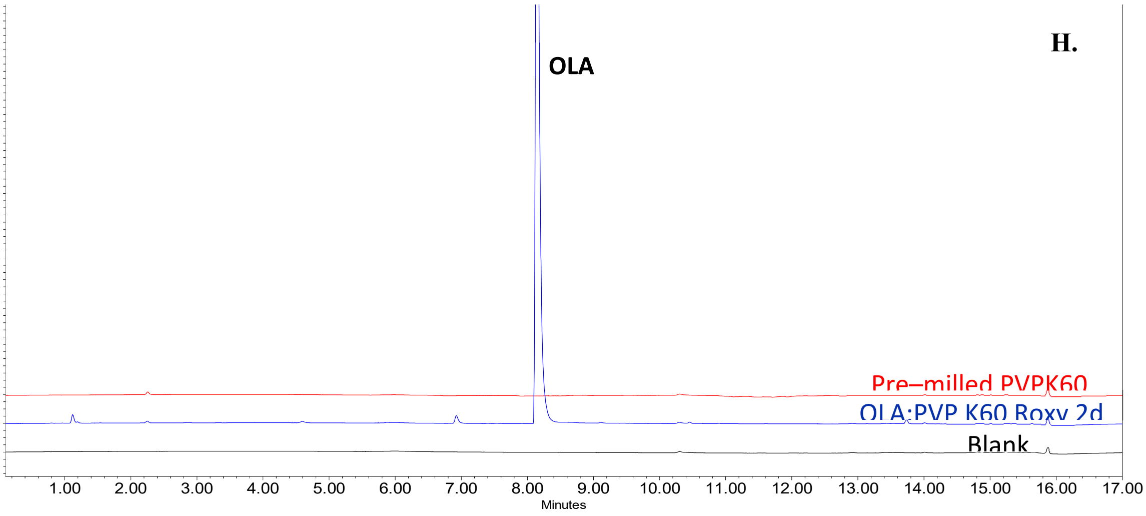
OLA
H.
Pre–milled PVPK60
OLA:PVP K60 Roxy 2d
Blank
1.00 2.00 3.00 4.00 5.00 6.00 7.00 8.00 9.00 10.00 11.00 12.00 13.00 14.00 15.00 16.00 17.00
Minutes

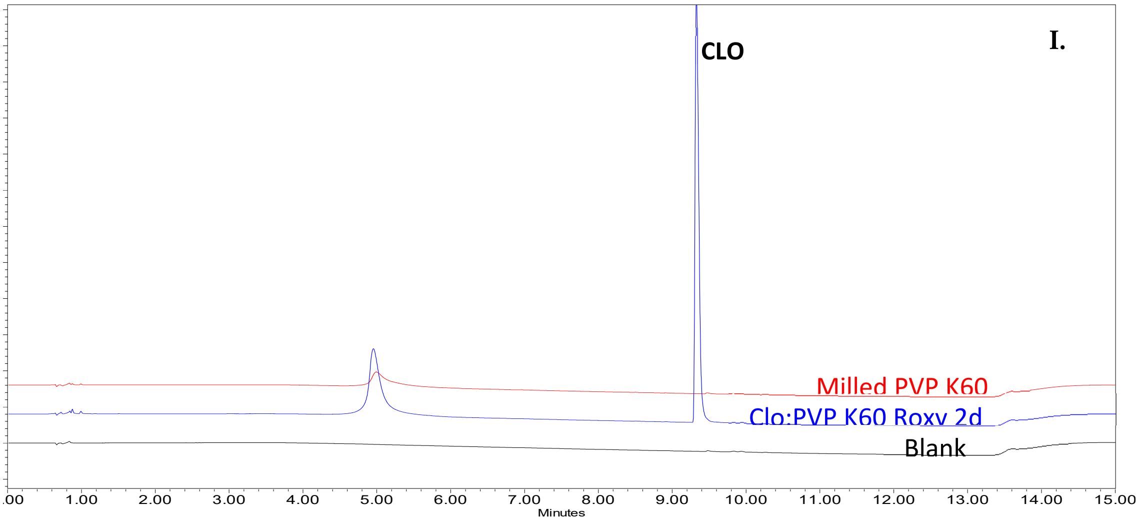

Figure S2. Overlaid chromatograms of solid state oxidation stress on the selected drug candidates with pre–milled PVP K–60 (Roxy indicates RapidOxy®)

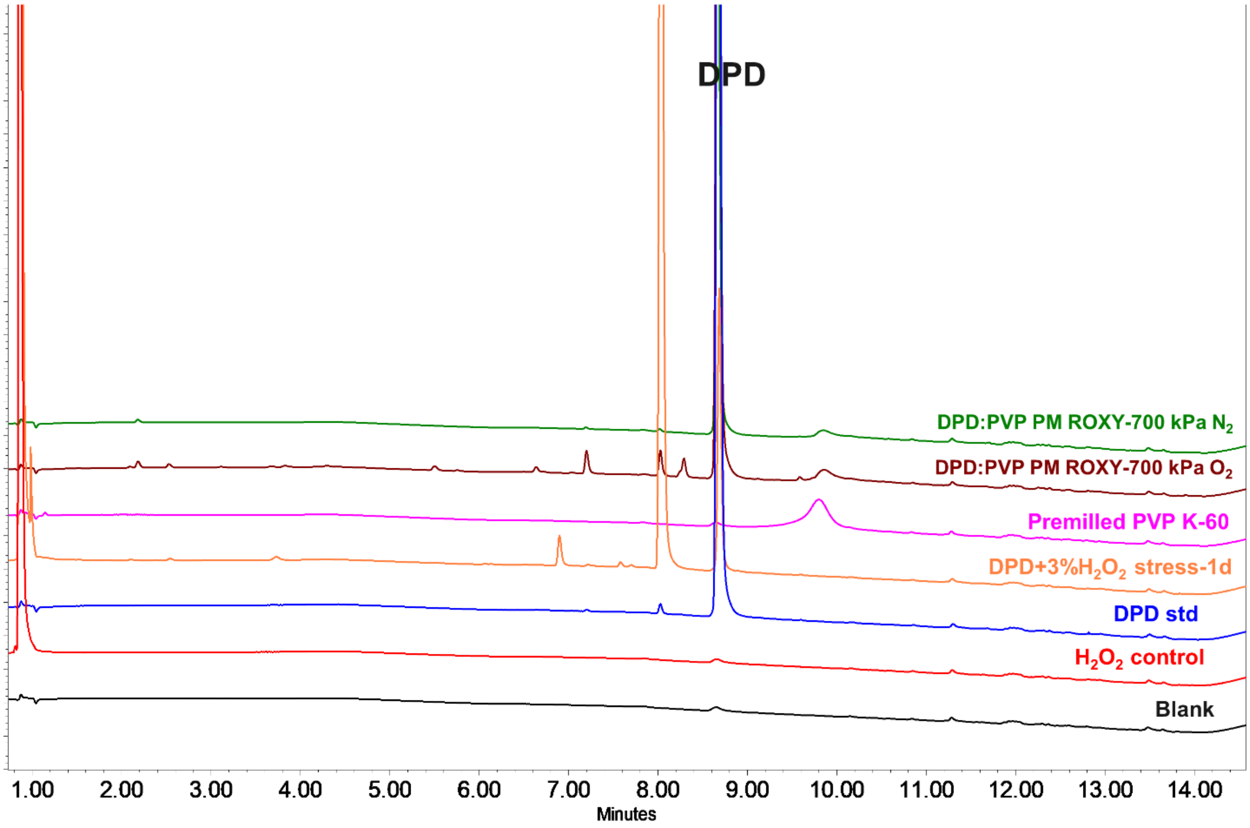

Figure S3. Control experiments under elevated nitrogen pressures (in RapidOxy®) and solution stress experiment using H_2O_2. (std–standard; PM–physical mixture; ROXY–RapidOxy)

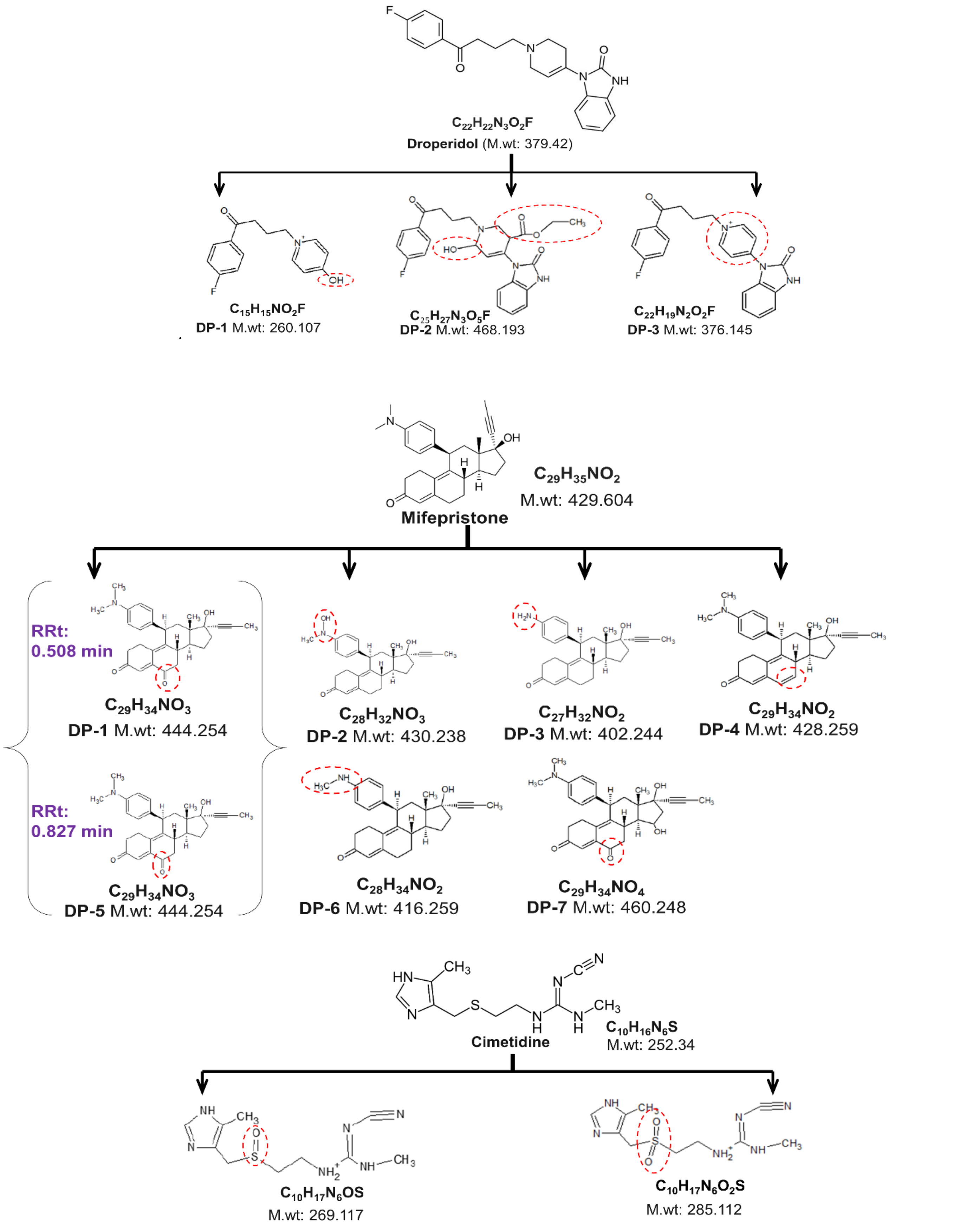

Figure S4. Structures of DPs formed in Drug:PVP PM (1:10) under RapidOxy® conditions as characterised by LC–MS analyses.

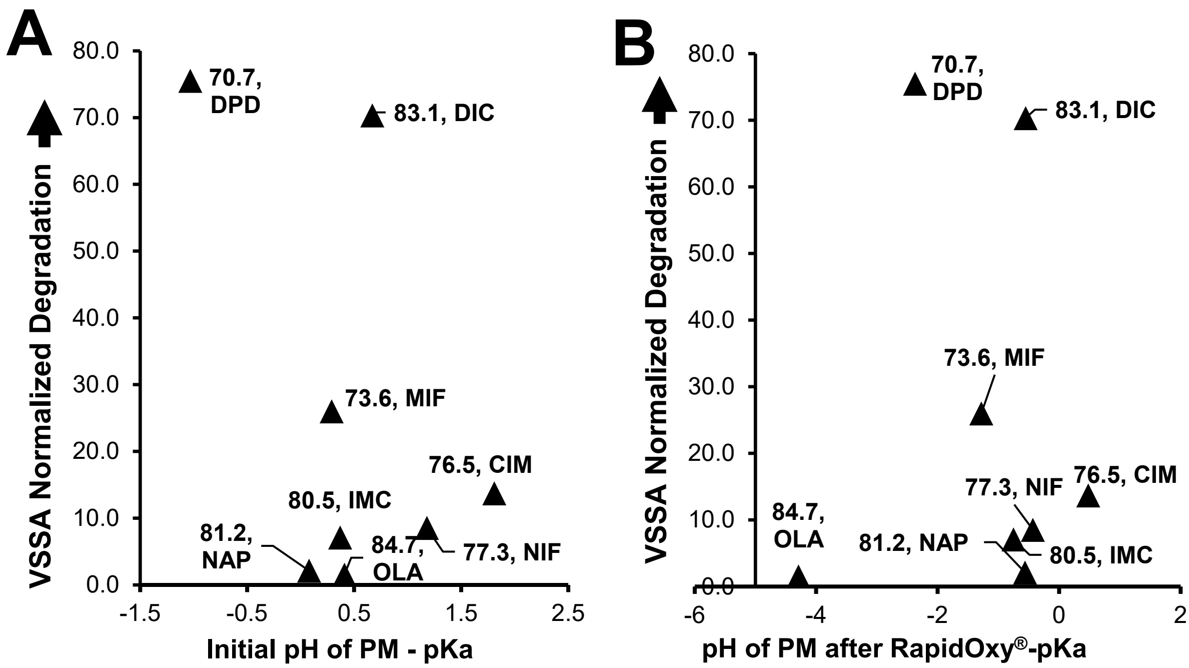

Figure S5. Comparison of extent of ionization (pH–pKa) and the VSSA normalized degradation in the solid state drug:premilled PVP K–60 (1:10) mixture before (A), and after RapidOxy® exposures (B). *PM stands for Physical mixture and VSSA is the volume specific surface area.
